# Retraction: Combined application of zinc and iron-lysine and its effects on morpho-physiological traits, antioxidant capacity and chromium uptake in rapeseed (*Brassica napus* L.)

**DOI:** 10.1371/journal.pone.0272189

**Published:** 2022-08-03

**Authors:** The PLOS ONE Editors

The *PLOS ONE* Editors retract this article [1] because it was identified as one of a series of submissions for which we have concerns about authorship, competing interests, and peer review. We regret that the issues were not addressed prior to the article’s publication.

IEZ, SA, MHS, HSY, AM, ZA, and MR did not agree with the retraction. MHA, AA, and XW either did not respond directly or could not be reached.
